# The Study of Reactive Ion Etching of Heavily Doped Polysilicon Based on HBr/O_2_/He Plasmas for Thermopile Devices

**DOI:** 10.3390/ma13194278

**Published:** 2020-09-25

**Authors:** Na Zhou, Junjie Li, Haiyang Mao, Hao Liu, Jinbiao Liu, Jianfeng Gao, Jinjuan Xiang, Yanpeng Hu, Meng Shi, Jiaxin Ju, Yuxiao Lei, Tao Yang, Junfeng Li, Wenwu Wang

**Affiliations:** 1 Key Laboratory of Microelectronics Devices & Integrated Technology, Institute of Microelectronics, Chinese Academy of Sciences, Beijing 100029, China; lijunjie@ime.ac.cn (J.L.); liujinbiao@ime.ac.cn (J.L.); gaojianfeng@ime.ac.cn (J.G.); xiangjinjuan@ime.ac.cn (J.X.); huyanpeng@ime.ac.cn (Y.H.); shimeng@ime.ac.cn (M.S.); tyang@ime.ac.cn (T.Y.); lijunfeng@ime.ac.cn (J.L.); wangwenwu@ime.ac.cn (W.W.); 2 Advanced Sensing Department, Wuxi Internet of Things Innovation Center Co. Ltd., Wuxi 214001, China; 3 School of Science, China University of Geosciences, Beijing 10083, China; liuhao1398@cugb.edu.cn; 4 College of Electronic and Information Engineering, North China University of Technology, Beijing 100144, China; zhangj@ncut.edu.cn (J.J.); 16152010908@mail.ncut.edu.cn (Y.L.)

**Keywords:** heavily doped, polysilicon, etch, HBr/O_2_/He, thermopile

## Abstract

Heavily doped polysilicon layers have been widely used in the fabrication of microelectromechanical systems (MEMS). However, the investigation of high selectivity, anisotropy, and excellent uniformity of heavily doped polysilicon etching is limited. In this work, reactive ion etching of undoped and heavily doped polysilicon-based hydrogen bromide (HBr) plasmas have been compared. The mechanism of etching of heavily doped polysilicon is studied in detail. The final results demonstrate that the anisotropy profile of heavily doped polysilicon can be obtained based on a HBr plasma process. An excellent uniformity of resistance of the thermocouples reached ± 2.11%. This technology provides an effective way for thermopile and other MEMS devices fabrication.

## 1. Introduction

Polysilicon, including both undoped and heavily doped polysilicon, has many important applications in complementary metal oxide semiconductor (CMOS) and micro electro mechanical systems (MEMS) technologies [1,2,3,4,5,6,7]. Compared to undoped polysilicon, heavily doped polysilicon has higher Seebeck coefficient, lower electrical resistivity and thermal conductivity. Therefore, heavily doped polysilicon has attracted wide research interest for applications of thermoelectric power generators [8,9]. In thermopile devices, heavily doped polysilicon is used as thermocouple material due to its CMOS-compatibility and perfect thermoelectric performance.

For thermopile devices fabrication, it is of great significance to develop techniques with high reproducibility, high yield, and excellent uniformity for fabricating a number of micro or nanostructures with various shapes and dimensions. Therefore, the manufacturing of anisotropy and high selectivity with respect to etching of masking materials and underlying layers of etching heavily doped polysilicon has become an important technology.

In the past years, anisotropic etching of undoped polysilicon has been extensively studied. Fluorine-containing plasmas can be used to etch polysilicon at low substrate temperatures and pressures. Nevertheless, selectivity over SiO_2_ is insufficient in F-based plasmas [10,11]. Instead, bromine-containing plasmas are used for polysilicon etching because spontaneous etching by bromine atoms is very slow, as is ion-assisted etching of SiO_2_. Thus, there has been increasing interest in HBr-based plasma because of improved selectivity with respect to the photoresist mask and SiO_2_, as well as superior control of the etched sidewall profile [10,12,13,14]. Although successful results of polysilicon etching have been obtained based on HBr plasmas, most of them are undoped polysilicon. In contrast, anisotropic etching of heavily doped polysilicon is limited studied, and most of them are based on fluorine- and chlorine-containing plasma [15,16]. How to accurately control the profile of heavily doped polysilicon and improve selectivity is still a challenging subject.

In this article, we focus on the etching of undoped, heavily doped n-type and p-type polysilicon based on a HBr plasma. The profile of undoped and heavily doped polysilicon after etching are studied by high resolution scanning electron microscope (HRSEM). Plasma-surface interactions occurring during over etching of three types of polysilicon with high-density HBr/O_2_/He plasmas have been investigated by x-ray photoelectron spectroscopy (XPS). The purpose of this article is to describe a high selectivity, anisotropy, and excellent uniformity process suitable for heavily doped polysilicon etching which could be used for thermopiles or other MEMS devices fabrication in the future.

## 2. Materials and Methods

**Figure 1:**
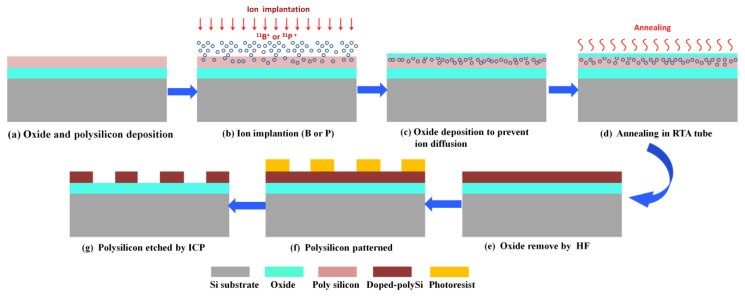
Schematic diagram of the process for fabricating heavily doped polysilicon strip structures.

All the materials were performed on 8-inch (100) silicon wafers (Global Wafers Corporation, Taiwan). Figure 1 schematically illustrates the preparation process of the heavily doped polysilicon strip. 

Step 1: The process started with depositing of plasma enhanced chemical vapor deposition (PECVD) of oxide and low pressure chemical vapor deposition (LPCVD) of polysilicon films.

Step 2: Polysilicon layers were implanted respectively with boron and phosphorus as the condition shown in Table 1.

Step 3: To prevent the dopants out from diffusion in the following activation procedure, a screen oxide of a 20 nm layer was deposited by plasma enhanced chemical vapor deposition (PECVD) before annealing.

Step 4: The heavily doped wafers were treated at 1050 °C for 60 s in N_2_ ambient by rapid thermal annealing (RTA).

Step 5: The screen oxide film was removed by 100:1 diluted hydrofluoric acid (DHF) for about 10 min, thus leaving heavily doped polysilicon film to be patterned by lithography. 

Step 6: Photoresist lattices with a diameter of 10 μm were fabricated on the wafer surface by 365 nm I-line lithography.

Step 7: The patterned polysilicon samples were etched in Lam TCP9400 (Lam Research Corporation, Fremont, CA, USA), an inductively coupled reactor.

The samples were etched in mixed HBr/O_2_/He plasma with the condition shown in Table 2. In order to increase physical bombardments, helium gas was added. The etching process employed here consists of three different steps. Breakthrough was the first step set up for removing the thin native oxide on the polysilicon surface. Then main etch step was used to etch most of the portion of the polysilicon film controlled by an endpoint setup. In plasma containing a mixture of gases, the intensity of the spectra generated by a particular species was dependent on a number of factors, including its partial pressure (concentration) and the power applied to generate the plasma. As a particular gas began to make up an increasing portion of the plasma, the intensity of its spectral lines would also increase. Conversely, as a gas began to disappear from the plasma, the intensity of its spectral lines would decrease. The endpoint of the main etch of polysilicon was detected by monitoring 503 nm SiBr optical emission spectrum during processing. Therefore, the endpoint signal was obtained when the intensity of the spectral line decreased. After the main etch of polysilicon, the over etch step was generally required to clear the residual polysilicon layer on the wafer, and thus the over etch step was critical for reducing the underlayer oxide loss and also for control of the final etched profiles.

The morphology of the polysilicon patterns after etching was characterized by scanning electron microscope (SEM, Hitachi S-5500, Hitachi Corporation, Tokyo, Japan). X-ray photoemission spectroscopy (XPS) was employed to analyze the composition and the chemical bonds of the resultant. The XPS tool was ESCALAB 250Xi provided by Thermo Fisher (Thermo Fisher, MA, USA).

## 3. Results

### 3.1. Study of Undoped and Heavily Doped Polysilicon Etching 

The etch rate of oxide has a value of 70 Å/min under main etch, whereas the etch rate of oxide decreases to 10 Å/min under over etch. Therefore, much higher selectivity could be obtained during the over etch step compared to the main etch step. The lower SiO_2_ etch rate is attributable to the larger binding energy (191.1 kcal/mol) of the Si–O bond relative to that (78.1 kcal/mol) of the Si–Si bond [17]. There is no effective chemical mechanism for the removal of oxygen from the SiO_2_ surface. Previous studies of HBr reactive ion etching have suggested that the presence of carbon in the reactor has a strong influence on the SiO_2_ etch rate and the SiO_2_ etching can be enhanced by carbon contamination [18,19]. Accordingly, the decrease in the SiO_2_ etch rate with increasing O_2_ concentration under over etch results from the decrease in the amount of carbon in the reactor due to CO or CO_2_ formation.

**Figure 2:**
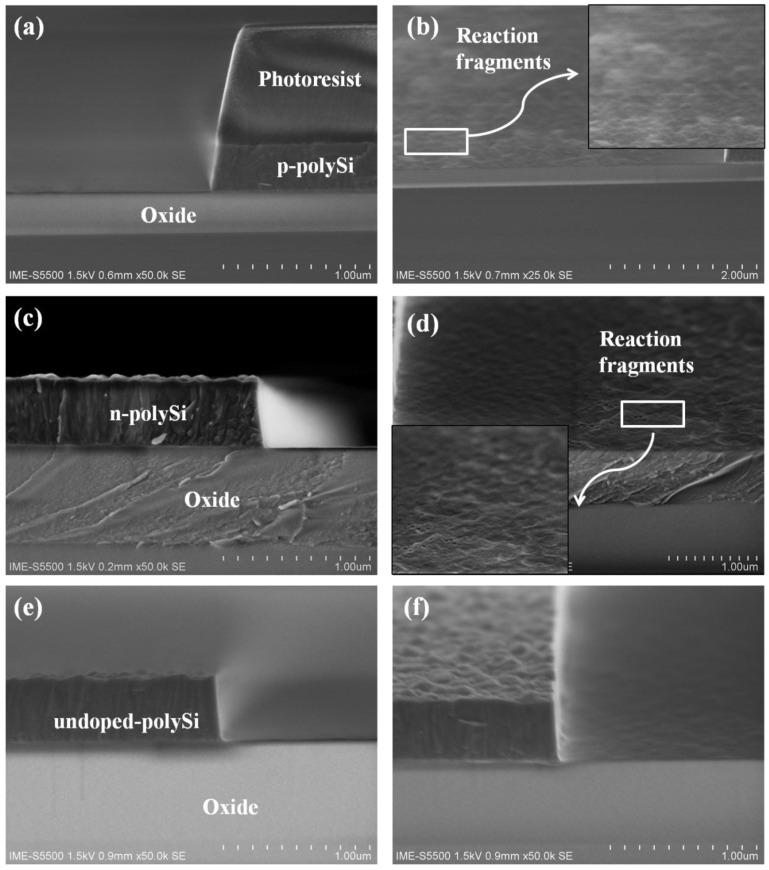
SEM images after etching of three types of polysilicon: (**a**,**b**) p-type polysilicon after etching, (**c**,**d**) n-type polysilicon after etching, (**e**,**f**) undoped polysilicon after etching. Images in (**a**,**c**,**e**) are the cross section views of polysilicon structures. Images in (**b**,**d**,**f**) are taken with a tilting angle of 20°.

The morphological results of the undoped and heavily doped polysilicon are shown in Figure 2. The etching conditions are given in Table 2. The main etch time are 139, 120, and 130 s obtained by endpoint for p-type, n-type, and undoped polysilicon, respectively. The duration of the over etch time of three types of polysilicon are set the same. It is seen that anisotropic profiles of undoped and heavily doped polysilicon could be obtained by using HBr/O_2_/He plasma. The sidewall angles of the three types of polysilicon strips are about 83°. However, quite different etching results are displayed between the undoped and heavily doped polysilicon. As can be observed in Figure 2b,d, some reaction fragments are formed at the interface between the polysilicon and oxide layer after n-type and p-type polysilicon etching. In contrast, the bottom of the undoped polysilicon structure after etching is much smooth without any residual.

### 3.2. Study of O_2_ Effect and Reaction Fragments

**Figure 3:**
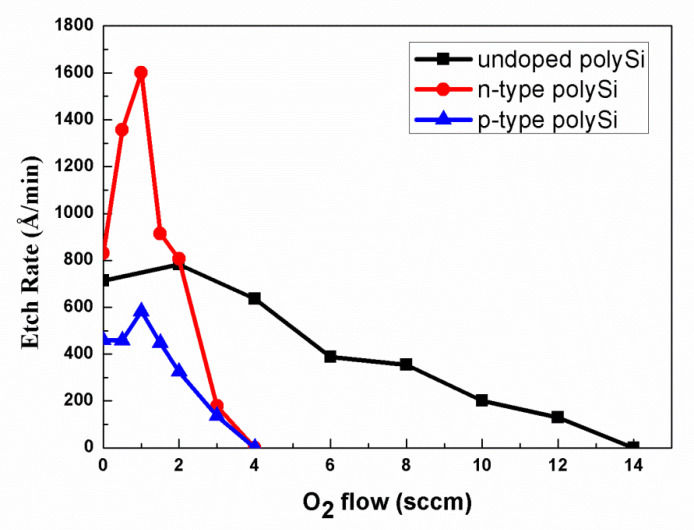
The etch rate of three types of polysilicon under different O_2_ flow.

Considering that the etch rate of the main etch step is not remarkably different between the three types of polysilicon according to the endpoint time, the reaction fragments of heavily doped polysilicon are assumed to be formed in the over etch step. As stated in the previous work, O_2_ concentration would affect the etch rate of undoped polysilicon [19,20]. Therefore, we verified whether the oxygen concentration affects the etching rate of heavily doped polysilicon in the over etch step. Figure 3 shows etch rates of heavily doped and undoped polysilicon as a function of O_2_ concentration in HBr/O_2_/He plasma. The other parameters of over etch are not change. The etch rate of undoped polysilicon has a value of 714 Å/min in HBr/He plasma without O_2_. Then, the etch rate of undoped polysilicon first increases and decreases gradually when O_2_ is added to HBr/He plasma. The observation is consistent with the previous study by Kow-Ming Chang et al. [10,20] on the etching mechanisms of polysilicon in halogen-based plasmas. For low O_2_ concentration, the polysilicon etch rate is controlled by the arrival rate of Br ions to the surface responding to an increase in the Br ion concentration in the plasma. The increase in the concentration of Br ions is similar to the situation for F ions in CF_4_/O_2_ plasmas, in which the F concentration increases at first (up to about 20% O_2_ addition) [10,21]. For high O_2_ concentration, the polysilicon etch rate decreases. The result may be due to the formation of a deposited silicon oxyhalide film (i.e., SiO_x_Br_y_) formed by a reaction between the etching products and the added oxygen on the interior surface of the etcher. The film will protect against the halogen radicals from surface recombination. Therefore, excessive addition of oxygen will oxidize the polysilicon and lead to a drop in the etching rate. 

An interesting phenomenon is that the etch rate of heavily doped n-type and p-type polysilicon first increases rapidly to a peak value at O_2_ concentration of 1 sccm and then deceases abruptly to zero at O_2_ concentration of 4 sccm. The etch rate of heavily doped polysilicon seem more sensitive of O_2_ concentration increment than that of undoped polysilicon. Still, it is puzzling that n-type and p-type polysilicon under identical etching conditions behave quite differently since the major atoms to be removed in both cases are silicon atoms and not dopants. For n-type polysilicon, at the O_2_ concentration at a low level, the etch rate is higher than either p-type or undoped polysilicon. When O_2_ concentration increases to 2 sccm, the etch rate of n-type polysilicon decreases equally to that of undoped polysilicon. As the O_2_ concentration continuingly increases, the etch rates of these two types of heavily doped polysilicon abruptly decline to zero. It is speculated that the reaction fragments formed affect the etching of heavily doped polysilicon.

**Figure 4:**
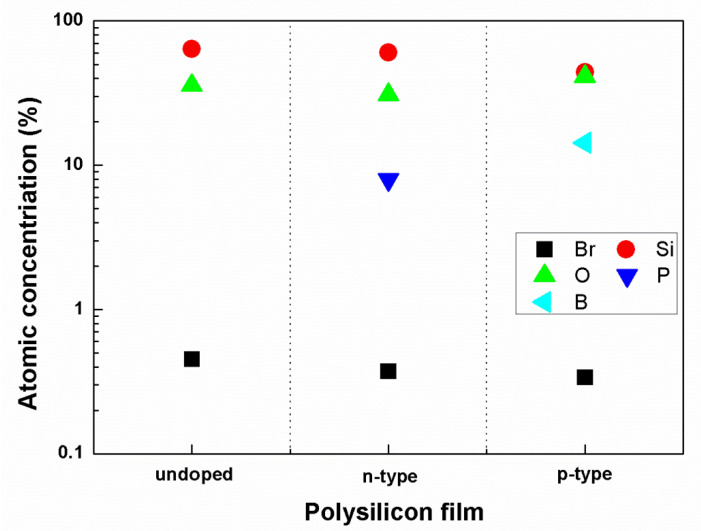
The compositions of three types of polysilicon film after etching measured by XPS.

In order to analyze the composition and the chemical bonds of the reaction fragments created after undoped and heavily doped polysilicon are etched, the etched surfaces of the three types of polysilicon film were characterized by XPS. Figure 4 gives the compositions of the reaction resultant of different polysilicon films after etching. It can be seen that the major components are Si, O, B, and P. Compared to these atoms, a small amount of Br is contained in the resultant film. The concentration of Br is about 0.45%, 0.37%, and 0.34% for undoped, n-type, and p-type polysilicon film, respectively. It indicates the resultant of the SiBr_x_O_y_ layer with a composition of nearly SiO_2_. This SiBr_x_O_y_ layer is assumed to compose the reaction fragment and stop the heavily doped polysilicon etching, as shown in Figure 2. Furthermore, the concentrations of B and P are about 14.3% and 7.9%, and this result will be analyzed in the following part.

**Figure 5:**
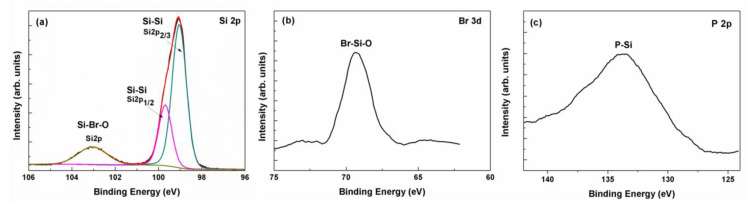

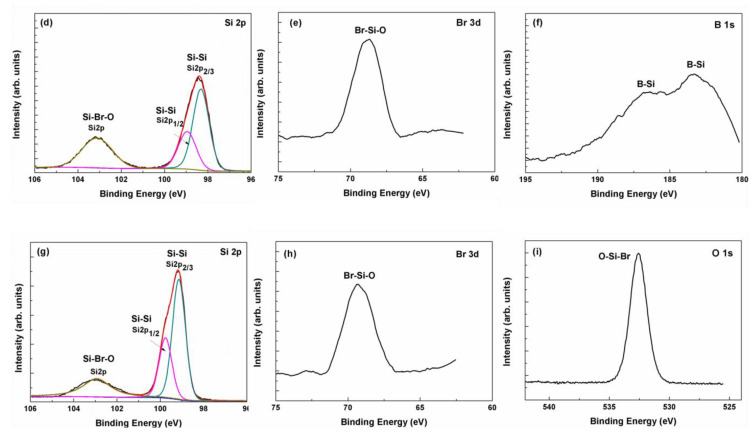
XPS spectra of (**a**,**d**,**g**) Si 2p; (**b**,**e**,**h**) Br 3d; (**c**) P 2p; (**f**) B 1s; (**i**) O 1s. (**a**–**c**) n-type polysilicon, (**d**–**f**) p-type polysilicon, and (**g**–**i**) undoped polysilicon.

Figure 5 shows XPS spectra obtained from doped and undoped samples that have been etched by HBr/O_2_/He plasma. The Si 2p spectrum of (a), (d), (g) are separated into two components: an elemental Si 2p spectrum with a binding energy of 99.4 eV and a chemically shifted Si 2p peak at a binding energy of 102.9 eV. Figure 5 b, e and h show the Br 3d spectra of three types of polysilicon. The peaks observed at 69 eV are attributed to the Br species adsorbed on the polySi surfaces. Furthermore, the binding energy of P 2p and B 1s electrons are 133, 183, and 186 eV, respectively. The results indicate that P-Si and B-Si bonds are formed on the heavily doped polysilicon wafer [22]. O 1s spectra are found in the three types of polysilicon and the peak value is 532 eV, as shown in Figure 5i. Moreover, the reaction fragments correspond to polysilicon and a reaction layer, respectively, which are formed on the polysilicon surface by plasma exposure. The signal around 102.9 eV of Si 2p indicates the reaction layer on the polysilicon surface is mainly attributed to a Si–Br–O oxide (SiBr_x_O_y_). This is because binding energies of SiBr_x_ (x = 1, 2, 3, 4) have values of 100.05, 100.7, 101.35, and 102.0 eV, respectively [23]. The peak intensity at 102.9 eV is smallest at the undoped polysilicon surface and largest at p-type polysilicon which is doped by boron ions. The SiBr_x_O_y_ layer with a composition of nearly SiO_2_ prevents the polysilicon from being etched because there is no effective chemical mechanism for the removal of oxygen from SiO_2_ surfaces. Therefore, the heavily doped polysilicon, especially the boron doped sample, is stopped from etching as the O_2_ concentration increases.

### 3.3. Study of Heavily Doped Polysilicon Etching Mechanism

**Figure 6:**
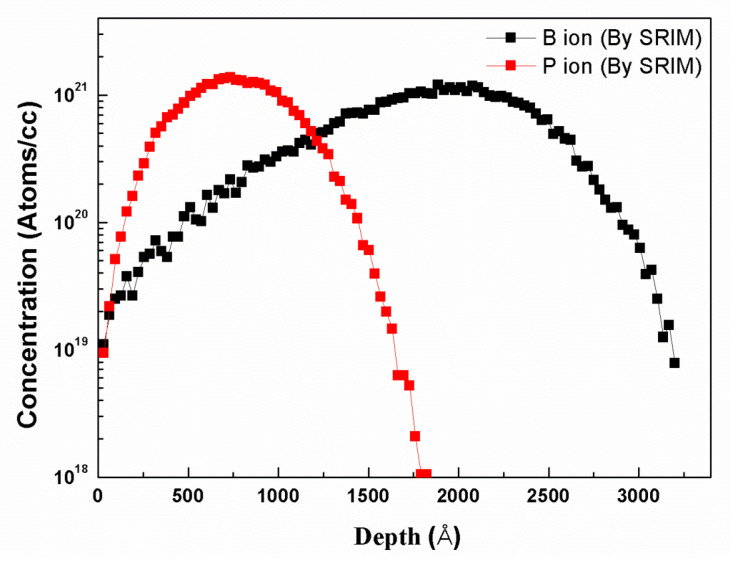
Simulation of B and P ion distribution by energy of 50 keV and dose of 1 × 10^16^ cm^−2^ in polysilicon.

In order to further illustrate the difference of the etching mechanism between the n-type and p-type polysilicon, the distribution of phosphorus and boron ion was simulated by SRIM [24]. The computation uses the statistical Monte Carlo method, which is based on the use of random numbers. The choice of this method is justified by the fact that the process of ion implantation has a statistical nature. In other words, the trajectories of the ions are determined by the interactions of the implanted ions with the target atoms and the final position of an implanted ion is where it has lost its complete energy. As seen in Figure 6, boron ions are more widely distributed than phosphorus ions. This means that the lighter ions (boron) travel deeper into the target than the heavier ions (phosphorus). Therefore, the concentration of boron ion in the reaction fragment is much more than the concentration of phosphorus ion. Moreover, phosphorus ion implantation might create more disordered target atoms and defects due to its heavier mass than boron ion, which will result in more ion scattering in the damaged region and limit the channeling effect of the implanted ions in the substrate.

**Figure 7:**
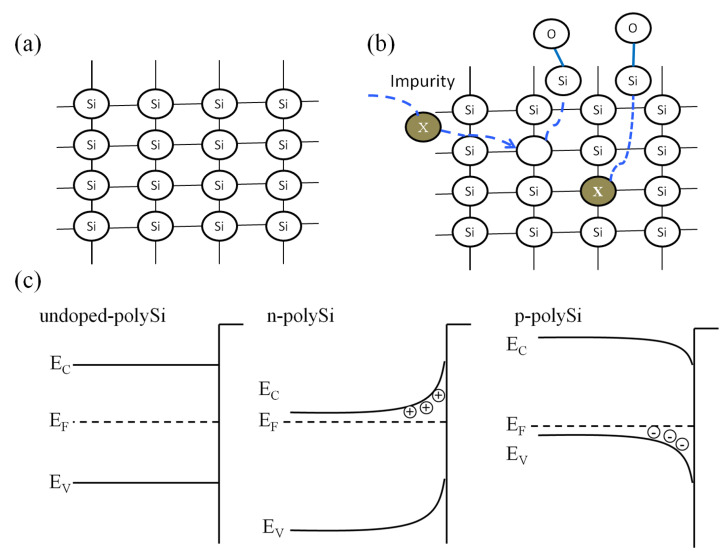
(**a**,**b**) Polysilicon lattice structure before and after ion implantation at a short range. (**c**) Schematic model for the doping effect during reactive plasma etching. Coulomb attraction in n-type and the Coulomb repulsion in p-type polysilicon play a major role in the field enhanced diffusion of a bromine atom.

For undoped polysilicon, the silicon lattice is ordered at a short range, as shown in Figure 7a. The diffusion mechanism of impurity atom has two kinds: interstitial diffusion and vacancy diffusion. The diffusion of boron and phosphorus atom is dominated by vacancy diffusion. Therefore, impurity atoms would occupy the location of the original silicon atoms in the lattice, and silicon atoms will stay in the interstice of the lattice. This kind of “free” silicon atom is assumed to be easily combined with oxygen to form a thin silicon oxide layer, as shown in Figure 7b. When the oxygen concentration is low, the formed silicon oxide layer is thin, the bromine atom can penetrate this thin layer to continue the process.

The different behavior of heavily boron and phosphorus doped polysilicon could be explained by the space charge. The opposite polarity of the space charge in the depletion layer induced by the Fermi level pinning is described in Figure 7c, with the presence of a negatively charged bromine atom on the silicon surface. The bromine atom could easily capture electrons supplied by the RF discharges because of the strong electron affinity. The Coulomb attraction between the uncompensated donor and the negative bromine pulls the bromine into the Si lattice, consequently increasing the etch rate in n-type silicon, whereas the Coulomb repulsion between the uncompensated acceptor and negative bromine pushes the halogen away from the Si lattice, leading to decrease in the etch rate in p-type polysilicon. Therefore, when the O_2_ concentration is low, the etch rate of undoped polysilicon is lower than that of n-type polysilicon, but higher than that of p-type polysilicon. However, when O_2_ concentration increases, the formed silicon oxide layers of heavily doped p-type and n-type polysilicon are thicker due to the “free” silicon atom. The bromine atom cannot penetrate this thick layer, leading to the reaction termination.

### 3.4. Material Structure and Resistance Measurement

**Figure 8:**
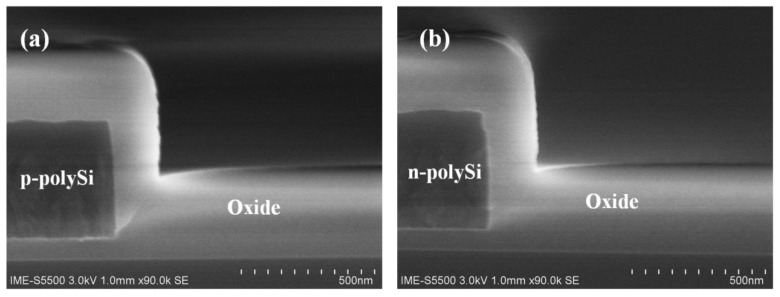
(**a**) p- type polysilicon and (**b**) n-type polysilicon covered with oxide film after etching.

According to the analysis, an optimized etching recipe which changed the O_2_ concentration to 1 sccm in the over etch step is used to etch heavily doped n-type and p-type polysilicon. After etching, the profile of heavily polysilicon is displayed on a smooth surface as well as undoped polysilicon. The underlayer oxide film is nearly without any loss. Subsequently, the slightly tapered sidewalls are conformally coated with a 300 nm thick PECVD silicon oxide layer, as shown in Figure 8.

**Figure 9:**
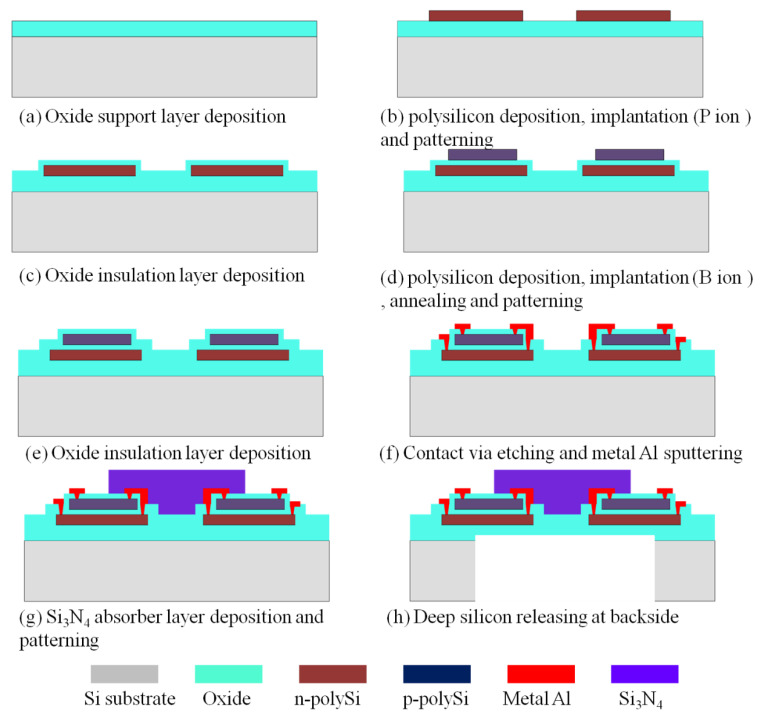
Schematic diagram of the thermopile fabrication process.

**Figure 10:**
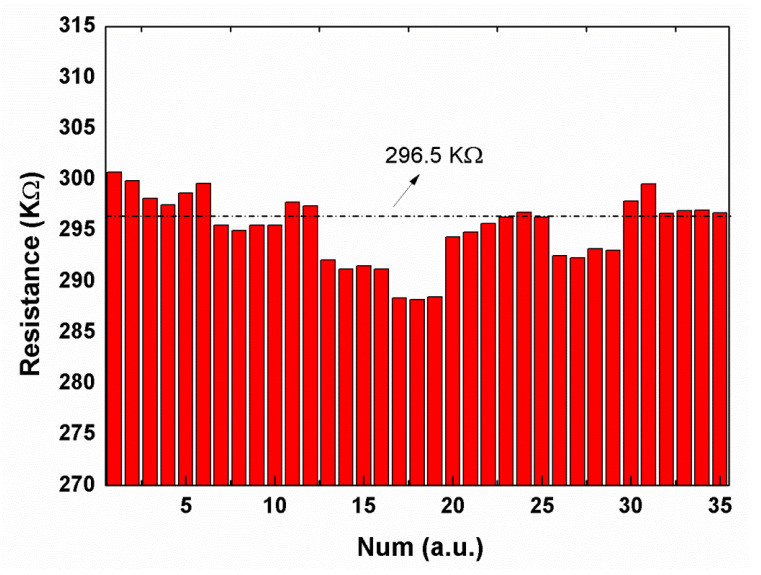
The resistance of thermopile devices tested by 1 V voltage.

The etching method of the heavily doped polysilicon is used to produce thermocouple structures for thermopile fabrication. The schematic diagram of the thermopile fabrication process is shown in Figure 9. Figure 10 shows the resistance of 35 thermopile devices. The designed resistance of the thermopile device is 296.5 KΩ according to the formula:

(1) $$R=N\sum_{i=1}^{2}\gamma_{i}\frac{l_{i}}{d_{i}\times w_{i}}$$

where R is the resistance of thermopile, N is the number of thermocouple strips, γ is the resistivity of the device, l, d, and w is length, thickness, and width of polysilicon, respectively (i = 1 for the p-type thermocouple strips; i = 2 for the n-type thermocouple strips). As shown in Figure 9, the maximum and minimum resistances of these devices are 300.7 and 288.2 KΩ, respectively. The uniformity of resistance of these devices is ±2.11%. Therefore, it is reasonable to use this etching method to obtain highly uniformity thermopile devices since the resistance of the thermocouple is directly related to the implantation concentration of the ion and morphology of etching.

## 4. Conclusions

In this work, the etching mechanism of undoped and heavily doped polysilicon based on a high density HBr/O_2_/He plasma is investigated. The effect of oxygen on the undoped and heavily doped polysilicon etching in HBr-based reactive ion etching plasmas is studied. The undoped polysilicon etch rate decreases gradually from 714 Å/min to zero as the O_2_ concentration increased from zero to 14 sccm. In contrast, O_2_ has a strong effect on the heavily doped polysilicon etching. The heavily doped n-type and p-type polysilicon etch rates rise first and then decrease abruptly from 1601 and 582 Å/min to zero, respectively. The abrupt decrease of the heavily doped polysilicon etch rate at relatively high O_2_ concentrations closely connects with the SiBr_x_O_y_ layer formed during the HBr/O_2_/He plasma exposure. Our results reveal that a low concentration of O_2_ in the HBr-based plasma is suitable for etching heavily doped polysilicon. The process is used to produce thermocouple strips and displaces a high uniformity of resistance after device fabrication. It is suggested that the method is a valuable candidate to etch a heavily doped polysilicon strip for thermopile or another MEMS device.

## Figures and Tables

**Table 1:** Injection and annealing conditions of polysilicon.

PolySi Type	Impurity Source	Dose (cm^−2^)	Energy (KeV)	Annealing Condition	Sheet Resistance (Ω/sq)
N-poly	PH_3_	1.0 × 10^16^	50	1050 °C, 60 s	40
P-poly	BF_3_	1.0 × 10^16^	50	1050 °C, 60 s	60

**Table 2:** Etch conditions of polysilicon.

Step	Pressure (mTorr)	Source Power (W)	Bias Power (V)	Gas (sccm)	Time (s)
Breakthrough	10	350	−100	100 CF_4_	10
Main Etch	6	350	−100	110 HBr/1 O_2_/50 He	Endpoint detection
Over Etch	80	250	−240	150 HBr/4 O_2_/120 He	180
